# Chronic diseases among paramedics and their impact on mental health

**DOI:** 10.1192/j.eurpsy.2023.931

**Published:** 2023-07-19

**Authors:** I. Sellami, A. Feki, N. Remadi, N. Kotti, M. L. Masmoudi, K. Jmal Hammami, M. Hajjaji

**Affiliations:** 1 occupational medecine; 2 rheumatology, Hedi Chaker Hospital, University of Sfax, Sfax, Tunisia

## Abstract

**Introduction:**

Working in a care setting is characterised by an increased mental and physical load. During their professional life, personnel in this sector can develop essentially degenerative pathologies, which could influence their professional career as well as their psychological balance.

**Objectives:**

We aimed to evaluate the impact of chronic pathologies on the prevalence of anxiety and depression among this group.

**Methods:**

We conducted a cross-sectional study in hospitals in Sfax using a self-administered questionnaire. This questionnaire evaluated socio-demographic, professional, and clinical characteristics as well as an evaluation of the degree of anxiety and depression by the HAD questionnaire.

**Results:**

Our population consisted of 120 participants. The average age was 37 years, with a female predominance (a sex ratio of 0.69). The chronic pathologies found in the participants were mainly diabetes (18%), high blood pressure (4%), and rheumatic disease (6.7%). The average anxiety score was 8.18**±** 3.5 and that of depression was 9.02**±** 3.5. Certain depressive and anxious signs were found in 28.4% and 23.6% of participants, respectively. Although the average scores for anxiety and depression were higher in the subgroup of personnel with chronic pathologies (respectively, 9.8 versus 9.04 and 9.3 versus 8.46), these differences were not statistically significant (p > 0.05).

**Conclusions:**

Physical and mental health are both important to ensure a balanced life. Having good control of somatic illness can improve mental health.

**Disclosure of Interest:**

None Declared

